# Editorial: Application of Machine Learning in the Diagnosis of Dementia

**DOI:** 10.3389/fneur.2022.860607

**Published:** 2022-06-09

**Authors:** Kaoru Sakatani, Görsev Yener

**Affiliations:** ^1^Department of Human and Engineered Environmental Studies, Graduate School of Frontier Sciences, The University of Tokyo, Tokyo, Japan; ^2^Faculty of Medicine, Izmir University of Economics, Izmir, Turkey

**Keywords:** machine learning, dementia, blood test, MRI, NIRS (near infrared reflectance spectroscopy), MMSE, deep learning, diagnosis

This editorial aims to gather works on the application of Machine/Deep Learning methods on data spanning from widely used cognitive test, the MMSE, volumetry measurements of MRI, Time-resolved Near-infrared Spectroscopy (NIRS), and Basic Blood Test Data in diagnosis of incipient dementia.

In the paper by Syaifullah et al., “*Machine Learning for Diagnosis of AD and Prediction of MCI Progression From Brain MRI Using Brain Anatomical Analysis Using Diffeomorphic Deformation*,” the authors searched whether AI supports clinicians to diagnose or predict progression of dementia in patients with complaints of memory disturbance or suspected dementia. BAAD's SVMs outperformed two radiologists (90.5% vs. 57.5 and 70.0%). For patients with clinically suspected AD, the SVMcog support AD diagnosis with 95% accuracy, based on MRI and MMSE. Even in cognitively normal patients, ~90% are Aβ-positive when classified on the AD spectrum by SVMst. Therefore, these patients should be closely monitored for 3 years or more, and molecular PET examinations will be considered for DMT applications in the future. The current model may bear a potential for less invasive AD diagnosis with a low cost.

In another MRI ML study by Nanni et al., titled “*Comparison of Transfer Learning and Conventional Machine Learning Applied to Structural Brain MRI for the Early Diagnosis and Prognosis of Alzheimer's Disease*,” the authors aimed to evaluate the potential of deep-learning techniques (pretrained on generic images and then transferred to structural brain MRI) for the early diagnosis and prognosis of AD, with respect to conventional machine-learning approaches. Specifically, more than 600 subjects were obtained from the ADNI repository, including AD, Mild Cognitive Impaired converting to AD (MCIc), Mild Cognitive Impaired not converting to AD (MCInc), and cognitively-normal (CN) subjects. They evaluated the performance of the SVM-based classification method and four deep networks for AD diagnosis and MCI conversion prediction. These four networks (i.e., AlexNet, GoogleNet, ResNet, and Inception-v3) were pretrained on natural images and then transferred to structural brain MRI. Then, conventional classification methods, such as SVM plus feature extraction/selection, were used for comparison against pretrained networks. Their results indicated improved classification performance for discrimination of MCIc-vs-MCInc that increased the mean percentage AUC from 69.1 to 73.3. This uptrend is not replicated during other comparisons (AD vs. CN, MCIc vs. CN). Yet, this learning approach was able to effectively discriminate AD from CN with 90.2% AUC, MCIc from CN with 83.2% AUC, and MCIc from MCInc with 70.6% AUC, showing comparable or slightly lower results with respect to the fusion of conventional-ML systems.

In the study titled “*Machine Learning-based Assessment of Cognitive Impairment Using Time-Resolved Near-Infrared Spectroscopy and Basic Blood Test*” by Oyama and Sakatani, the authors have studied whether machine learning can predict cognitive function (i.e., the MMSE scores) in aged people using time-resolved near-infrared spectroscopy (TNIRS) to measure absolute concentrations of hemoglobin and optical path length at rest in the bilateral prefrontal cortices, basic blood test data, and age data. Then, they compared predicted MMSE scores and grand truth MMSE scores (mean MMSE scores = 22.9 ± 6.1) on 250 participants (mean age = 73.3 ± 12.6 years). Prediction accuracies were evaluated using mean absolute error (MAE) and mean absolute percentage error (MAPE). The DNN-based prediction using the blood test data alone exhibited the prediction accuracy with 81.8% sensitivity and 91.3% specificity (*N* = 202, 5-fold cross validation). The difference in MAPE between TNIRS and blood data was only 0.3%, and adding TNIRS data to the blood test data of the input layer only improved MAPE by 1.0% compared to the use of blood test data alone. The authors concluded that the DNN model using blood data with low cost and large-scale screening possibility may be suitable for mass screening.

In the study of Sakatani et al., titled “*Deep Learning-Based Screening Test for Cognitive Impairment Using Basic Blood Test Data for Health Examination*,” the authors aimed to develop a new screening test of cognitive impairment employing a deep neural network (DNN) to predict cognitive function (i.e., the MMSE score) based on subject's age and basic blood test (23 items). This method is based on the idea that cognitive impairment in the elderly is caused by systemic metabolic disorders such as lifestyle diseases, and thus, uses basic blood test data of health examinations. The study included 202 patients (73.48 ± 13.1 years) with various systemic metabolic disorders for training of the DNN model, and three other groups (*n* = 267) to validate the accuracy of the DNN model: a patient group on rehabilitation after stroke (*n* = 65, 73.6 ± 11.0 years), healthy participants attending a sports gym (*n* = 37, 62.0 ± 8.6 years), and a healthy examination group who visited the outpatient clinic of dementia prevention (*n* = 165, 54.0 ± 8.6 years). All subjects underwent the Mini-Mental State Examination (MMSE). The blood test data including basic complete blood count and metabolic panel were put into the input layer of the DNN model. The MMSE scores were put into the output layer of the DNN model as the ground truth data. They also evaluated the relationship between the blood test data and changes in MRI variables. As a result, significant strong positive correlations were noted between the predicted MMSE scores and ground truth scores in the Patient and Healthy groups (*r* = 0.66, *p* < 0.001). Furthermore, the predicted MMSE scores and ground truth scores in the patient group were similar; however, in the Healthy group, the predicted MMSE scores were slightly lower than the measured MMSE scores. They also reported that several blood test items significantly correlated with the variables on MRI variables. They concluded that this method may contribute to early detection of dementia and to personalized care for the prevention of it. Finally, they proposed an algorithm as combination of the DNN-based screening test and the MMSE followed by MRI.

Overall, this special issue may help to propose an algorithm for early detection of incipient dementia that may be helpful in the prevention of dementia and in health economics by means of DNN methods using (1) widely available basic blood tests, (2) MMSE scores, (3) MRI measurements, and (4) privacy issues related to personal data management.

## Author Contributions

Both authors confirm being contributors of this work and have approved it for publication.

## Conflict of Interest

The authors declare that the research was conducted in the absence of any commercial or financial relationships that could be construed as a potential conflict of interest.

## Publisher's Note

All claims expressed in this article are solely those of the authors and do not necessarily represent those of their affiliated organizations, or those of the publisher, the editors and the reviewers. Any product that may be evaluated in this article, or claim that may be made by its manufacturer, is not guaranteed or endorsed by the publisher.

